# Done with a degree? Immigration-specific disparities among holders of bachelor’s degrees in the transition to graduate studies in Germany

**DOI:** 10.3389/fsoc.2023.1204164

**Published:** 2023-10-12

**Authors:** Sebastian Neumeyer, Irena Pietrzyk

**Affiliations:** ^1^Leibniz-Institute for Educational Trajectories (LIfBi), Bamberg, Germany; ^2^Institute of Sociology and Social Psychology (ISS), University of Cologne, Cologne, Germany

**Keywords:** educational inequality, graduate education, immigrant background, educational aspirations, application, transition

## Abstract

In many Western societies, immigrants make more ambitious educational choices than their native counterparts of equal academic achievement and social origin. These ambitious decisions have been mainly observed at early and middle educational stages, whereas research on choices within higher education is scarce. Against this background, we investigate whether immigrants make more ambitious decisions than natives do also after having graduated from bachelor’s programs in Germany. We theoretically derive that variations in immigration-specific differences in educational choices can be expected based on social origin and country of origin, as well as between the application for and the actual enrollment in graduate studies. Using survey data on educational trajectories of bachelor’s degree holders, we observe our expectations to be confirmed for the investigated sample. First, immigration-specific differences in educational choices vary by social origin and are increased for graduates from low social origins. This finding supports that immigrants strive for status maximization, an idea that we understand as a theoretical specification of the motive for status gain. Second, they vary by country of origin, which suggests cultural factors to be subordinate. Third, immigration-specific differences in applications are more pronounced than differences in actual transitions, indicating that immigrants have fewer chances of transforming their aspirations into actual transitions. We conclude by discussing these three aspects more broadly.

## 1. Introduction

In many European countries, researchers have found strong disparities in terms of educational achievement and attainment between natives and immigrants (Heath et al., 2008). However, considering their lower levels of social origin and academic performance, immigrants lean more toward ambitious educational tracks. Researchers have found support for this phenomenon in early and middle educational stages in many Western countries (Fekjær and Birkelund, 2007; Kristen et al., 2008; Kristen and Dollmann, 2010; Jackson et al., 2012; Tjaden and Scharenberg, 2017; Dollmann, 2021; Busse and Scharenberg, 2022; Neumeyer et al., 2022). In the following, we refer to the phenomenon of immigrants making more ambitious educational choices compared to natives of equal social origin and academic performance as the *immigration-specific difference in educational choice*. This phenomenon is also known as the immigration-specific secondary effect (Heath and Brinbaum, 2007) and the positive choice effect (Dollmann, 2017).

However, it is not sufficiently clear whether immigrant students also make ambitious educational decisions after they have obtained their first university degree. The highest educational stage considered mainly in empirical studies has been the transition to university, with scarce research on later educational pathways. For Germany, two studies analyzed transitions within higher education. Neumeyer and Pietrzyk (2019) found no evidence for more ambitious educational choices of immigrants at the transition to doctoral programs. The study, however, did not differentiate between the intention for continuing education and the actual enrollment in PhD programs, a differentiation that might be especially important for pursuing a PhD due to strong performance-related selection by others. Jungbauer-Gans and Lang (2019) found that graduates with foreign citizenship who received their higher education qualification in the host country aspire more often to enroll in a graduate program than native graduates do, whereas immigrant graduates with a German citizenship do not. However, the authors did not empirically take into account possible variations based on country of origin. Further, strong performance-related external selection at advanced educational stages might attenuate the results on educational aspirations if the focus lies solely on enrollment. Therefore, the question of whether immigrant students are also highly ambitious within universities remains unanswered.

We help closing this research gap by investigating immigration-specific differences in educational choices after students have received their first university degree, i.e., whether differences between native and immigrant holders of bachelor’s degrees in their educational choices exist, considering social origin and academic performance. We examine this research question for Germany, which is a particularly interesting case. In Germany, the higher education system is choice-driven with low hurdles to enrollment, as almost no institutions charge tuition fees. Furthermore, graduate enrollment rates after having obtained a first degree are comparably high (*cf.* see Tienda and Zhao, 2017; Wakeling and Laurison, 2017). In such choice-driven systems, immigration-specific differences in choices tend to be particularly pronounced (*cf.* Griga and Hadjar, 2014), since for many immigrant students these systems open up the possibility of transferring their high aspirations into corresponding choices.

In our study, we contribute more broadly to research on immigration-specific disparities in education by applying three differentiations. First, we pay attention to variations based on *social origin*—thereby stimulating theory development. Researchers mostly explain immigrants’ ambitious choices by their strong desire for upward mobility (e.g., Kao and Tienda, 1995; Vallet, 2007). However, this determination has not been further specified. Thus, it is unclear what distance from parents in terms of socioeconomic positioning immigrants strive for. Investigating immigrants from low social origins at late educational stages can help clarify this question. After all, these persons who themselves graduated from bachelor’s programs would most likely experience transgenerational mobility already after having obtained their first university degree. If these individuals still made more ambitious choices than their native counterparts did, their motivation could be *status maximization* against the background of already accomplished educational successes—a theoretical concept that we understand as a specification of the motivation for status gain. Whereas previous research confirmed the importance of an interaction between immigrant status and social origin on low and middle educational stages (e.g., Dollmann, 2017), no studies investigated educational choices of immigrants from low social origins within higher education so far.

Second, we examine variations of immigration-specific differences in educational choices by *country of origin*. Previous research established country-specific variations in many Western countries (e.g., Dollmann, 2021; Rudolphi and Salikutluk, 2021). However, these studies did not consider some important immigrant groups. Based on unique data that provide high case numbers, we are able to differentiate between the largest immigrant groups in Germany, thereby not only providing information on immigrants with a background from Turkey and from the former Soviet Union, but also on immigrants with a background from further labor market recruiting countries and from Poland. The opportunities for comparison resulting from these differentiations may provide impulses for why some immigrant groups (do not) strive ambitiously for education.

Third, we distinguish between the *application* to graduate programs on the one hand and the actual *transition* on the other hand. This differentiation takes into account that transitioning to graduate studies is not only driven by the aspiration to continue higher education but also by external performance related obstacles. Further, it broadens the perspective on immigration-specific disparities in education as research so far mainly focused on actual transitions. By implicitly conceptualizing disparities in educational pathways as a sequence of various steps (for disparities based on social origin, see Roderick et al., 2011; Finger, 2016) this approach makes it possible to look deeper into difficulties that some groups of graduate students may face in transferring their aspirations into actual transitions. Hence, we analyze whether immigrants’ lower chances for translating aspirations into transitions may be associated with their lower academic performance level.

We investigate the questions of whether immigration-specific differences in educational choices exist and how they vary regarding the above-mentioned three important aspects based on a German-wide survey of university graduates. We make use of a unique data set with a large sample size that allows us to simultaneously consider various differentiations (*N* = 70,744 bachelor’s graduates from the classes of 2011–2014). We show that, also within higher education, some groups of immigrant graduates indeed have a stronger educational determination than their native counterparts of equal social origin and performance level in Germany, and that immigration-specific gaps in educational choices turn out differently based on the three differentiations.

## 2. Theoretical framework

### 2.1. Ambitious choices

Immigrant students’ educational pathways are characterized by two phenomena that work in opposite directions. Immigrant students usually have lower chances for transitioning to high-order tracks than their native counterparts of equal social origin due to, on average, lower levels of academic performance (for an overview, see Heath et al., 2008). This performance-driven disadvantage for immigrant students is termed the immigration-specific primary effect (Heath and Brinbaum, 2007), analogous to the primary effect of social origin (Boudon, 1974). Even though it is not finally clarified why native students outperform immigrants, it appears plausible that differences in the linguistic proficiency of the host country drive disparities in academic competencies across various domains if the starting conditions are particularly disadvantageous. In line with this thought, immigration-specific primary effects can be traced back to vocabulary skills in early childhood (Becker and Klein, 2021). However, the picture of immigration-specific disparities at transitions becomes more complicated when the comparatively lower academic achievement of immigrants (e.g., in terms of grades) is additionally considered. In this case, students with a family history of migration choose more ambitious educational tracks (Jackson et al., 2012; e.g., Dollmann, 2017; Tjaden and Scharenberg, 2017). Researchers termed this phenomenon the immigration-specific secondary effect (Heath and Brinbaum, 2007), again, analogous to the secondary effect of social origin (Boudon, 1974).

The fact that immigrant students make more ambitious educational choices than their native counterparts of equal social origin and performance level goes back to their comparatively high levels of educational aspirations. Researchers have proposed different explanations for this phenomenon (for an overview: Becker and Gresch, 2016). According to the immigration optimism hypothesis, which is the most influential explanation for this phenomenon, immigrant students might exhibit more determination for intergenerational status gain than their native counterparts (Kao and Tienda, 1995; Vallet, 2007). Since emigrating involves the high cost of leaving the familiar environment behind, persons who have voluntarily migrated are assumed to be positively selected in their desire for a better life and for upward mobility. This desire frequently does not translate into the desired socioeconomic situation in the host country, with first-generation immigrants often holding low positions. Therefore, education might be perceived as a crucial vehicle for upward mobility for descendants (Vallet, 2007, p. 142). Immigrants thus supposedly strive for intergenerational status gain through education. In contrast, native persons avoid status demotion with no particular desire for intergenerational upward mobility, according to rational choice theories modeling social class differences (Erikson and Jonsson, 1996; Breen and Goldthorpe, 1997; Esser, 1999). Previous research indeed finds empirical support for the immigration optimism hypothesis (e.g., Salikutluk, 2016; Dollmann, 2017; Tjaden and Hunkler, 2017).

Since the ambitious educational choices of immigrants reveal themselves most typically under consideration of academic achievement and social origin, the consideration of these background characteristics is crucial when dealing with the phenomenon of strong educational ambition. In Germany, parents’ educational attainment, as part of social origin, is the most important predictor of higher education pathways (Müller et al., 2017, p. 344), probably because formal education and labor market outcomes are strongly connected (e.g., Müller et al., 1998), and because the costs of education are comparatively low. Therefore, parents’ educational attainment can be reasonably used in Germany when considering the social origin of university graduates.^1^

Due to the general finding of a strong educational determination of immigrants, we expect immigrants who have received a bachelor’s degree to make more ambitious educational decisions than their native counterparts of equal social origin and performance level (*H1*).

### 2.2. Interaction with social origin

Of the explanations for ambitious educational decisions, the idea of status gain and status maintenance suggests an interaction between immigrant status and social origin. Before paying special attention to immigration-specific disparities, researchers focused on class differences in educational choices and proposed that all persons are motivated by avoiding status demotion (Erikson and Jonsson, 1996; Breen and Goldthorpe, 1997; Esser, 1999). Even though this motive is assumed to be equal across social groups, it is expected to play out differently for students depending on their social origin.

For example, deciding for or against higher education, persons with university-educated parents would risk status demotion if they did not attend university and would, therefore, most likely decide accordingly. Persons whose parents did not receive any professional training do not rely on higher education for maintaining their status and, therefore, would in many cases decide against university (see also Esser, 1999, p. 268). Developing this theoretical idea further, the pattern of class disparities can be expected to vary across different transitions. For example, most persons with university-educated parents will opt for university. In contrast, persons whose parents hold a PhD are also likely to pursue their doctoral studies based on their motivation for status maintenance (for empirical results, see Lörz and Seipelt, 2019).

The specific educational transition is also significant for whether immigration-specific disparities can be expected for groups with particular social origins based on motivations for status maintenance and status gain. Focusing on the decision to continue higher education after receiving a bachelor’s degree, at least four groups can be distinguished based on social origin and immigrant status.

Within the group of persons whose parents attended vocational training as the highest education level, native persons will frequently not continue their education since they already received a bachelor’s degree, guaranteeing status maintenance. In contrast, immigrants of equal social origin might seek further education based on their motivation for intergenerational status gain. Therefore, immigration-specific disparities for transitioning into graduate studies are very likely for students whose parents finished vocational training. From a theoretical perspective, these choices are particularly interesting. Individuals from low social origins who themselves graduated from bachelor’s programs would most likely experience some social mobility already after having obtained their first university degree. If immigrant graduates still strived more determinedly for further education at this educational stage than their native counterparts, their motivation could be *status maximization* against the background of already accomplished educational success—a theoretical idea that we understand as a specification of the motivation for status gain.

Among persons whose parents hold at least a master’s degree,^2^ no immigration-specific disparities can be expected based on different motivations. Both the motivation to avoid status demotion and the motivation for status gain imply continuing education after receiving a bachelor’s degree for this social origin group.

Previous research has indeed found the assumed pattern of interaction between social origin and immigrant status on early and middle educational stages (Dollmann, 2017; Sudheimer and Buchholz, 2021). However, no studies investigated interactions on later states or examined the choices of immigrants from low social origins within higher education in particularly —a focus potentially shedding light on the motivation for status maximization.

Against the background of our theoretical considerations on the interaction between immigrant status and social origin, we expect the immigration-specific difference in educational choices to be larger for holders of bachelor’s degrees from low social origins than for their peers from high social origins (*H2*).

### 2.3. Country-specificity

It is well known that immigration-specific differences in educational choices vary by country of origin. Previous research has revealed that it is particularly students with a background from Turkey who strive more strongly for education in Germany (Kristen et al., 2008; Relikowski et al., 2012; Salikutluk, 2016). In the Netherlands, Sweden, and Switzerland, similar patterns have been observed (Tjaden and Scharenberg, 2017; Dollmann, 2021; Rudolphi and Salikutluk, 2021). While researchers have thoroughly uncovered the high significance of country-specific variations in educational determination, the reasons behind these variations are far from well understood.

In Germany, persons of Turkish origin are the most visible minority group with high levels of perceived discrimination (Hans, 2010; Horr et al., 2020; Diehl et al., 2021). Therefore, students with a Turkish background might particularly seek to compensate for anticipated labor discrimination with high educational achievements. Additionally, these immigrants might have perceived the efforts and struggles of immigration as especially burdensome, leading to a strong positive selection based on the desire for upward mobility among this group. More specifically, the geographical distance between Germany and Turkey is high in comparison to the country of origin of some other large minority groups in Germany, such as persons from Poland. This circumstance might have been perceived as a serious challenge a few decades ago. Furthermore, the process of naturalization was more complicated for Turks than for persons from the former Soviet Union, another large minority group in Germany that often gained residency rights based on Jewish or German heritage.

In Germany, scientific research frequently did not consider all relevant immigrant groups due to insufficient data. However, in addition to persons of Turkish origin, which is the largest immigrant group in Germany, other immigration waves have resulted in other large immigrant communities in Germany. During the 1960s and 1970s, workers were recruited not only in Turkey but also in Italy, Greece, Portugal, Spain and in countries of the former Yugoslavia—all of these individuals were able to immigrate to Germany based on numbers agreed between Germany and the sending countries without having to meet additional requirements. These groups became significant in size as they grew with the descendants of the workers. Additionally, immigration from Poland and from countries of the former Soviet Union, largely beginning in the 1990s, led to large immigrant groups in Germany. Polish migrants have often been granted residence permits because of their German origin, while migrants from the former Soviet Union have been able to stay in Germany both because of their German origin and because they were Jewish. Following legal changes in the 1990s, the number of immigrants in Germany has declined significantly. Due to the heterogeneity of the mentioned groups in terms of, among other things, the time, reasons, and circumstances of their migration, as well as their social composition, we assume variations in the immigration-specific difference in educational choices across countries. Such variations may provide impulses on the reasons why some immigrant groups (do not) strive ambitiously for education. However, scarce research in this field limits specific predictions beyond the expectation that persons of Turkish origin are particularly ambitious.

Accordingly, we expect immigration-specific differences in educational choices to vary across different groups defined by country of origin, with a particularly large immigration-specific difference for holders of bachelor’s degrees with a Turkish background (*H3*).

### 2.4. Application and actual transition

Principally, not all universities provide enough places in their master’s programs for all their bachelor’s graduates of corresponding programs at the university level. In Germany, about 40% of master’s programs are subject to admission restrictions (Hochschulrektorenkonferenz, 2020). A small proportion of graduates leaves the university because they are unable to find a suitable master’s program or because they do not meet admission criteria (Alesi and Neumeyer, 2017). This external selection criterion for pursuing graduate education in master’s programs is mainly performance-related (Scheller et al., 2013). Access to master’s programs is even more linked to admission requirements than the access to bachelor’s programs (Winter et al., 2012).

Immigrants might experience greater difficulties in fulfilling these requirements and, therefore, in realizing their aspirations to continue with graduate education after obtaining a bachelor’s degree. The chance of admission might be mitigated by immigrant students’ comparatively low levels of academic performance (Neumeyer and Pietrzyk, 2019; Klein and Müller, 2020). In addition, immigrant graduates might have less information regarding admission requirements since they are, on average, less socially integrated into higher education (Schaeper, 2020).

Therefore, it is reasonable to distinguish between applications and actual enrollment when investigating immigration-specific disparities in education. Such a perspective that expands the standard focus on actual transitions makes it possible to look deeper into difficulties that some groups of graduate students may face in transferring their aspirations into actual transitions.

Against the outlined background, we expect the immigration-specific difference in educational choices to be stronger for application to than for actual enrollment in graduate programs by holders of bachelor’s degrees (*H4*).

## 3. Method

### 3.1. Data

To test our hypotheses, we apply data from the German Cooperation Project Tracer Studies (*Kooperationsprojekt Absolventenstudien* or KOAB), a nationwide online survey of graduates from higher education institutions.^3^ The KOAB data provide detailed measures of educational and professional careers after receiving a bachelor’s degree and a sufficient sample size to distinguish important characteristics. Compared to other datasets available in Germany, it most importantly provides information on country-specific immigrant status. Since graduates take part in the survey approximately 1.5 years after graduation, the time span is long enough to observe the transition into further programs.^4^ To gain a sufficient sample size for specific subgroups defined by country-specific immigrant status and social origin, we pool data from the graduating classes of 2011, 2012, 2013, and 2014.

The KOAB includes graduates of up to 68 higher education institutions each year. As institutions self-select into survey participation, the results can only be cautiously generalized nationwide. However, participating institutions are heterogeneous and represent the broad range of institutions in Germany quite well regarding, for example, type of institution, range of fields, prestige, size, age, and region. To tackle the underrepresentation of graduates from universities of applied sciences, female graduates, and foreign citizens, we apply weights (range 0.6–4.9) based on nationwide distributions for the respective graduate cohorts (Federal statistics office, 2021). Institutions invited all graduates from a given year to participate in the survey. Yearly response rates vary between 35 and 44%.

We included only graduates who obtained their higher education qualification in Germany for reasons of comparability (Kristen, 2014, p. 118), thereby excluding the so-called *Bildungsausländer* (foreigners in terms of education) who obtained their university entrance qualification abroad. Furthermore, we excluded two participating institutions from Austria, graduates who were not asked the relevant questions due to slight variations of the survey between institutions, and graduates older than 45 years.^5^

To handle item nonresponse, we multiply imputed data with iterated chained equations (White et al., 2011).^6^ The imputation models included all predictor and outcome variables of our analysis models as well as the interaction between immigrant status and social origin. Additionally, we included a small set of auxiliary variables that are associated with the level of model variables and/or missing values of model variables. Cases with any imputed outcome were not included in the final analyses (von Hippel, 2007). This left us with a sample of *N* = 70,744 graduates from 75 institutions. We imputed a total number of 30 imputations, which is sufficient based on the fraction of missing information in our most complex analysis models with interaction effects (von Hippel, 2020).

### 3.2. Measures

#### 3.2.1. Outcomes

We constructed dichotomous indicators for the outcomes. These are submitting an application for a study program and the transition to a study program within 1.5 years after graduation from a bachelor’s program.^7^

#### 3.2.2. Independent variables

We defined the *immigrant background* by parents’ country of birth. Therefore, our operationalization of the immigrant background includes persons of the first and the second generation without distinguishing between generation status.^8^ We distinguished between the four most common minority groups in Germany (i.e., Turkey; other labor market recruiting countries — Spain, Portugal, former Yugoslavia, Greece, Italy; countries of the former Soviet Union; Poland) and a residual country category. Graduates who have only one parent that was born abroad were included into the respective immigrant group. We excluded graduates with parents from different country categories (about 1% of all graduates with at least one parent born abroad). In the following analyses, we apply a dichotomous measurement of immigrant background regardless of country of origin and an operationalization that captures the country categories.

*Social origin* is measured by the parents’ highest vocational or academic degree. As our theoretical considerations are closely connected to the motives for avoiding status demotion and for upward mobility, we chose a differentiated operationalization. More specifically, we operationalized social origin in five categories, including (i) no degree, (ii) vocational education and training (VET), (iii) short-cycle higher-education (i.e., traditional degrees from universities of applied sciences or from engineering schools),^9^ (iv) long-cycle higher-education (equivalent to a master’s degree),^10^ and (v) a PhD.

When analyzing interaction effects between social origin and immigrant status, we focused on the second and the fourth level. These levels are directly below and above the bachelor’s degree and, therefore, provide clear hypotheses about educational aspirations based on the motives for status maintenance and for status gain. In the other educational groups, which are rather extreme, immigrants are strongly under- or overrepresented (Neumeyer and Pietrzyk, 2019, p. 447). This hampers the investigation of immigration-specific disparities due to small sample sizes. The two categories, which we focused on, account for around 75% of the sample (see Table 1). However, our results are largely robust to a more exhaustive operationalization of social origin by a dichotomous variable indicating that at least one parent has a higher education degree.^11^

**Table 1:** Frequencies by immigrant background and social origin (absolute numbers and column percentages).

Immigrant background	Natives	Turkey	Other labor market recruiting countries	Former Soviet Union	Poland	Other	Total
**Social origin based on parents’ educational attainment**							
1. No degree	247	637	298	116	30	326	1,654
	0.4%	46.9%	20.7%	3.6%	1.4%	8.9%	2.3%
2. Vocational education and training (*low* category)	28,669	535	772	1,407	1,396	1,318	34,098
	48.7%	39.4%	53.8%	43.3%	63.7%	36.0%	48.2%
3. Short-cycle HE	10,250	52	87	492	224	333	11,437
	17.4%	3.8%	6.0%	15.2%	10.2%	9.1%	16.2%
4. Long-cycle HE (*high* category)	15,765	115	223	1,140	488	1,334	19,066
	26.8%	8.5%	15.5%	35.1%	22.3%	36.4%	26.9%
5. Doctorate	3,920	18	57	93	51	351	4,490
	6.7%	1.3%	3.9%	2.9%	2.3%	9.6%	6.3%
Total	58,851	1,358	1,436	3,248	2,190	3,662	70,744
	100.0%	100.0%	100.0%	100.0%	100.0%	100.0%	100.0%

Weighted and imputed data. HE, Higher education.

#### 3.2.3. Controls

To estimate immigration-specific differences in educational choices, we control for *academic achievement* in school and in the bachelor’s program in all analyses. We operationalized school achievement by the z-standardized grade point average of the higher education entrance qualification. Correspondingly, academic achievement in the bachelor’s program is measured by the grade point average of the bachelor’s degree. To account for field-specific grading practices, we standardized the average grades within combinations of field of study and class cohorts. For an easier interpretation, we reversed all variables measuring academic achievement so that higher values indicate better grades. Additional checks revealed a nonlinear relationship between grade point average in the bachelor’s degree and the outcome variables, with a stronger association at low to intermediate achievement levels. Therefore, we included both a linear term and a squared term of the grade point average.

Enrollment in a graduate program and immigrant status are associated with the *type of institution and field of study* (e.g., Kristen et al., 2008; Neugebauer et al., 2016; Mentges and Spangenberg, 2021). To control for these associations, we employed a combination of both variables.

Descriptive information regarding the variable distributions is provided in Table 2.^12^

**Table 2:** Descriptive statistics on outcomes, independent variables, and controls.

	Share/mean	Standard deviation	Minimum	Maximum	Share imputed
**Application for a further study program**					
No	27.3%				
Yes	72.7%				
**Enrollment in a further study program**					
No	30.5%				
Yes	69.5%				
**Immigrant background**					
Native	83.2%				8.1%
Turkey	1.9%				
Other labor market recruiting countries	2.0%				
Former Soviet Union	4.6%				
Poland	3.1%				
Other	5.2%				
**Social origin**					
No degree	2.3%				11.3%
Vocational education and training	48.2%				
Short-cycle HE	16.2%				
Long-cycle HE	26.9%				
Doctorate	6.3%				
School GPA	0.01	1.00	−2.64	2.23	1.1%
Bachelor’s degree GPA	0.00	1.00	−4.74	2.68	5.5%
School GPA (raw)*	2.37	0.62	1	4	
Bachelor’s degree GPA (raw)*	2.05	0.49	1	4	
**Field of study**					
U Math/Sciences	10.9%				
U Engineering	8.7%				
U Computer Science	2.2%				
U Economics	7.0%				
U Humanities/Arts	8.8%				
U Social Sciences	4.4%				
U Educational Sciences	2.0%				
U Social Work	0.9%				
U Teaching	2.8%				
U Other	2.4%				
UaS Engineering	20.8%				
UaS Computer Science	3.0%				
UaS Economics	12.1%				
UaS Social Work	5.4%				
UaS Other	8.6%				
**Gender**					
Male	50.3%				2.1%
Female	49.7%				
**Year of graduation**					
2011	24.2%				
2012	23.7%				
2013	26.4%				
2014	25.7%				

Weighted and imputed data (*N* = 70,744). HE, Higher education; GPA, Grade point average; U, University; UaS, University of applied sciences. *Only unimputed values are reported.

### 3.3. Analytical procedures

To test our hypotheses, we conducted a set of logistic regressions. The application and the actual transition to graduate programs are treated as outcome variables. Immigrant background is the main independent variable. To estimate immigration-specific differences in educational choices, we continuously controlled social origin and achievement in all models.^13^

Based on these logistic regressions, we computed predicted probabilities for application and for the actual transition for different social groups. Furthermore, we computed the average marginal effects (AMEs) of an immigrant background, which quantify differences in percentage points (p.p.).

To test our hypotheses concerning differences in the immigration-specific differences in educational choices across different conditions, we compared the AMEs for different social groups and for different outcomes. We employed the procedure proposed by Mize et al. (2019), which allows us to consider the covariation between conditions. When testing for differences at various levels of social origin, we focused on two levels of social origin (i.e., vocational education and training and long-cycle higher education), while all levels were included in the estimation.

Furthermore, we tested the robustness of our results regarding variations in the analytical procedure (linear probability models; unweighted data; usage of complete cases only) and in the operationalization of independent variables (social origin: dichotomous operationalization based on whether at least one parent graduated from university; immigrant background: exclusion of graduates with only one parent that was born abroad). The results remain comparable (see Supplementary Figures S1, S2).

## 4. Results

First, we report immigration-specific differences in educational choices across different levels of social origin (i.e., only controlling for social origin; Section 4.1). Second, we illustrate how the immigration-specific difference in educational choices varies between different levels of social origin (Section 4.2). Within these subsections, we discuss variations of the immigration-specific differences based on country of origin and on whether they are investigated for the application or for the actual transition.

### 4.1. Differences in educational choices across different levels of social origin

**Figure 1:**
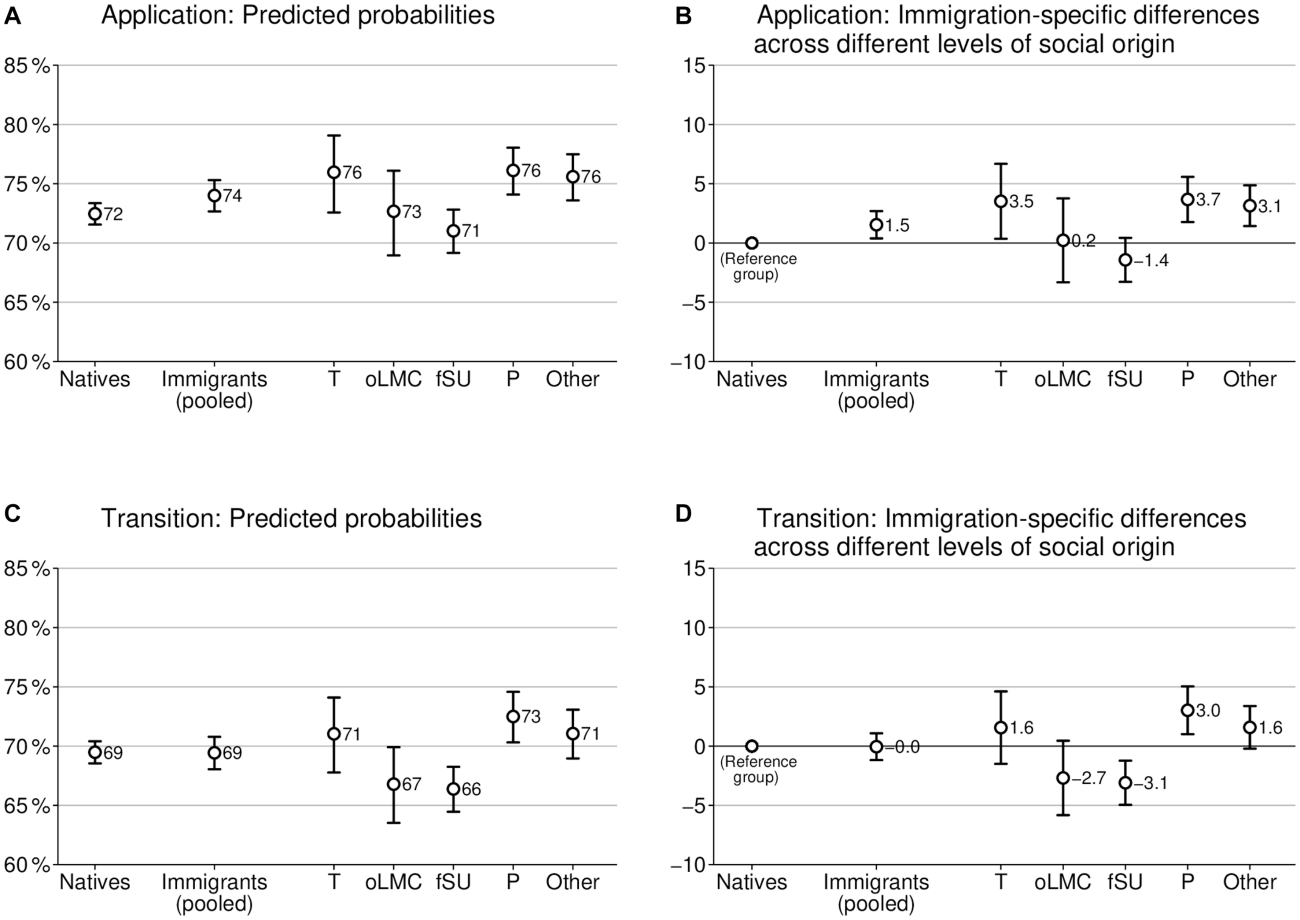
Predicted probabilities and immigration-specific differences in educational choices across different levels of social origin. The left column **(A,C)** shows predicted probabilities, and the right column **(B,D)** illustrates differences between immigrant and native bachelor’s graduates, based on logistic regressions in quantities of percentage point differences (AME × 100); 95% confidence intervals; controls: social origin, achievement, field of study, gender, and year of graduation. For full models: see Supplementary Table S7. Immigrants (pooled): dichotomous operationalization of immigrant background regardless of country of origin; T: Turkey; oLMC: other labor market recruiting countries; fSU: former Soviet Union; P: Poland; Other: other countries. *N* = 70,744.

The main results of our investigation on differences in educational choices across different levels of social origin are illustrated in Figure 1. The subplots at the top of Figure 1 summarize the results of the application, while the plots at the bottom provide information about the actual transition. The plots on the left side contain the predicted probabilities. The plots on the right side inform about differences between immigrant and native graduates in percentage points (p.p.), and therefore provide straightforward effect sizes for the immigration-specific difference in educational choice.

Looking at social groups defined by a dichotomous differentiation between graduates with and without an immigrant background (“Immigrants (pooled)”), we find immigrants to be more ambitious than natives regarding application (Figure 1B). More specifically, immigrant graduates apply to graduate programs approximately 1.5 p.p. more often than native graduates do, considering their social origin and achievement and additional controls. However, no immigration-specific difference exists in the actual transition to graduate studies (Figure 1D). This confirms our general hypothesis about more ambitious educational choices among immigrants (H1), but only for the outcome of applying to graduate programs. Therefore, this result underscores that immigration-specific differences in educational choices within higher education might be underestimated when the focus lies solely on the actual transition.

Comparing different outcomes, the immigration-specific difference in submitting applications is significantly higher than the difference in actual enrollment (*p* < 0.01). This is in line with hypothesis H4. The illustration of the predicted probabilities shows neatly (Figures 1A,C) that this difference between outcomes stems from varying rates of realizing educational aspirations across groups. The model predicts that 69% of native graduates enroll in a graduate program with an application rate of 72%. For immigrant students, the predicted value for enrollment is also 69%, but with a predicted application rate of 74% (all values under consideration of the social origin and achievement level). Therefore, immigrant graduates have less chance of transforming their applications into actual transitions to graduate studies than native graduates have.

Moving to variations in immigration-specific differences in educational choices based on country of origin, we see strong differences between countries of origin (Figures 1B,D). Graduates of three groups (Turkey, Poland, Other) make more ambitious educational choices than native graduates do, at least regarding one outcome, ranging up to 3.7 p.p. In contrast, graduates with a background from labor market recruiting countries other than Turkey or from a country of the former Soviet Union do not differ from native graduates or even display less ambitious educational choices. Against our expectations (H3), persons with a Turkish background are not generally more determined than other immigrant groups.

However, the previous finding of a lower probability of transforming applications into actual transitions into graduate programs also holds for all immigrant groups. For example, persons from other labor market recruiting countries are similar to native graduates in submitting applications, but they show a lower probability of enrolling. The AMEs for submitting applications are statistically significantly higher than the AMEs for transitions for most immigrant groups (oLMC, fSU, Other: *p* < 0.01; Turkey *p* < 0.05).

**Figure 2:**
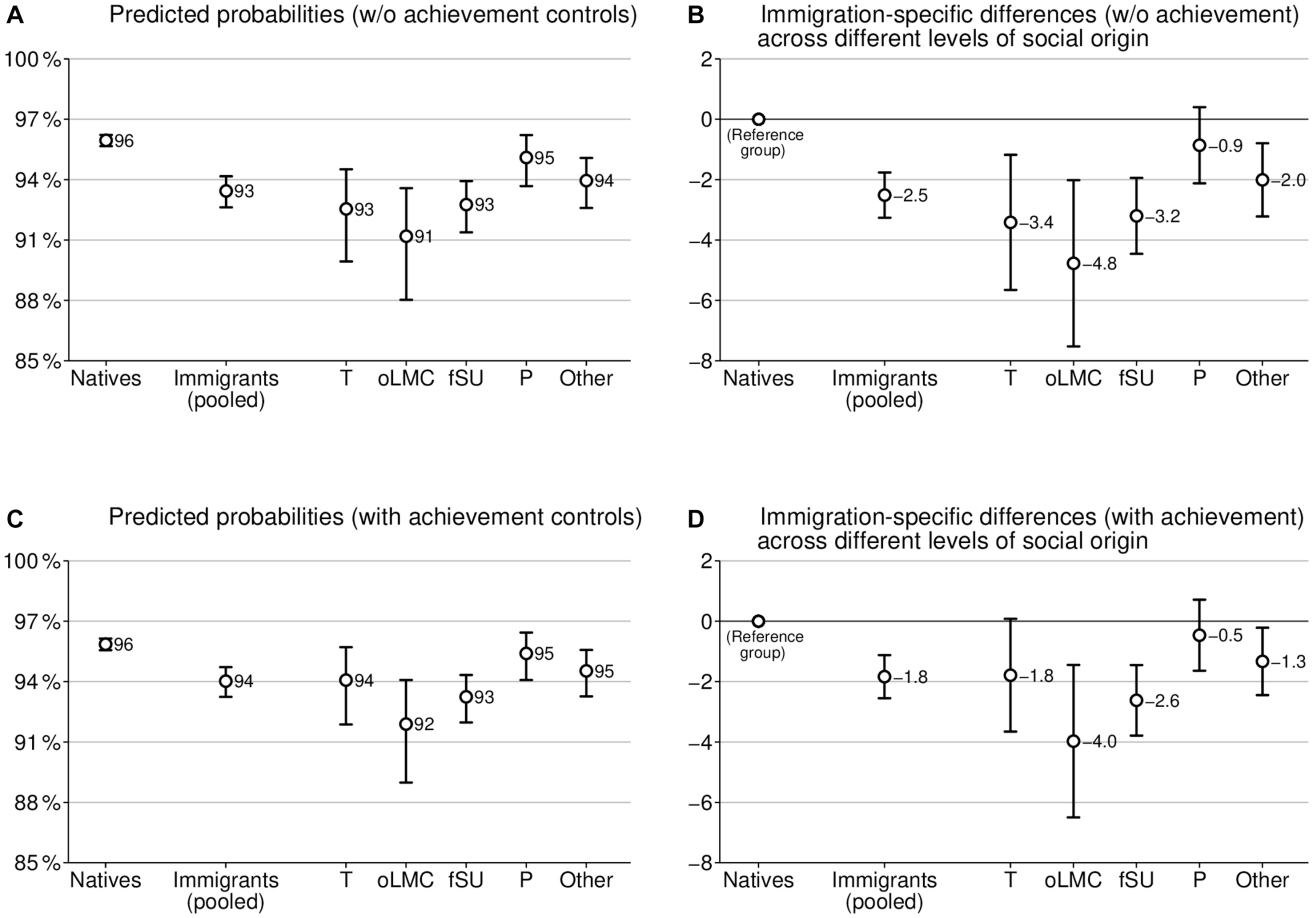
Predicted probabilities and immigration-specific differences in transition, conditional on application. The left column shows predicted probabilities **(A,C)**, and the right column **(B,D)** illustrates differences between immigrant and native bachelor’s graduates, based on logistic regressions in quantities of percentage point differences (AME × 100); 95% confidence intervals; controls: social origin, field of study, gender, and year of graduation; graphs **(C)** and **(D)** additionally control for achievement. Immigrants (pooled): dichotomous operationalization of immigrant background regardless of country of origin; T: Turkey, oLMC: other labor market recruiting countries, fSU: former Soviet Union, P: Poland, Other: other countries. *N* = 55,028.

To directly investigate the pattern of fewer chances for transforming applications into transitions among immigrant graduates when compared to their native counterparts, we conducted additional analyses. More specifically, we analyzed differences between immigrants and natives in transitions only for the subgroup of graduates who have applied to a program (see Figure 2). Using this procedure, we also observe lower transformation chances for most groups of immigrant graduates, which reaches up to an approximately 5 p.p. difference for graduates with a background from other labor market recruiting countries (Figure 2B). We tested whether these lower transformation chances are associated with performance differences by introducing academic achievement into the estimation (Figure 2D). The pattern of associations indeed suggests that differences in academic performance drive the lower chances for transformation, even though we are not able to test directly for a causal impact. However, performance differences cannot explain the whole pattern of lower chances. Therefore, factors beyond academic achievement contribute to the comparatively lower chance for transforming applications into enrolment among immigrant holders of bachelor’s degrees.

To summarize the results across different levels of social origin, we can observe immigrants to make slightly more ambitious educational choices than natives when the country of origin is not differentiated (H1). However, this immigration-specific difference exists only for submitting applications (1.5 p.p.) and not for actual enrollment, with the difference in applying being larger than in actual enrollment (H4). Furthermore, we see great variations between countries of origin (H3), with not only graduates with a Turkish background being particularly ambitious, but also graduates with a Polish background and a background from further countries. Furthermore, we observe fewer chances of transforming the application into actual enrollment for most immigrant graduates compared to natives when considering social origin and academic achievement.

### 4.2. Differences in educational choices in interaction with social origin

**Figure 3:**
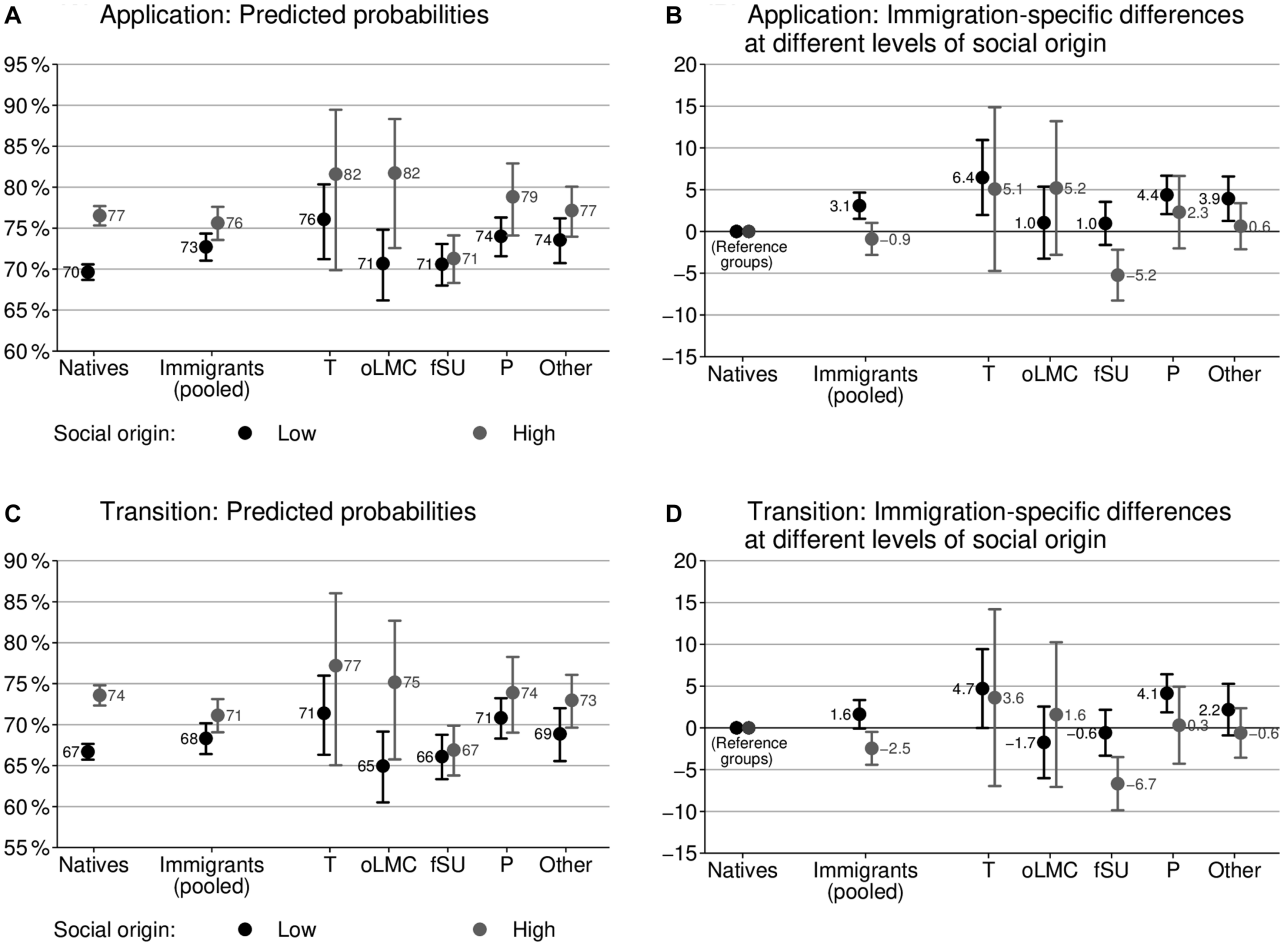
Predicted probabilities and immigration-specific differences in educational choices at different levels of social origin. The left column **(A,C)** shows predicted probabilities, and the right column **(B,D)** illustrates differences between immigrant and native bachelor’s graduates of the same social origin, based on logistic regressions in quantities of percentage point differences (AME × 100); 95% confidence intervals; controls: achievement, field of study, gender, and year of graduation. For full models, see Supplementary Table S8. Immigrants (pooled): dichotomous operationalization of immigrant background regardless of country of origin; T: Turkey; oLMC: other labor market recruiting countries; fSU: former Soviet Union; P: Poland; Other: other countries. *N* = 70,744.

The main results of our examination of immigration-specific differences in educational choices in interaction with social origin are illustrated in Figure 3. Again, the subplots differentiate between submitting an application and enrollment and illustrate the predicted probabilities and AMEs for immigration-specific differences in educational choices.

We first focus on immigration-specific differences in educational choices when utilizing a dichotomous differentiation between graduates with and without an immigrant background (Figures 3B,D). We can see that immigrant graduates from low social origins apply more frequently to graduate programs (3.1 p.p., *p* < 0.01) and slightly more often enroll in graduate programs (1.6 p.p., *p* > 0.05) than their native counterparts from low social origins of equal academic achievement. In contrast, immigrant graduates from high social origins apply slightly less often to graduate programs (−0.9 p.p., *p* > 0.05) and actually enroll less often in graduate programs (−2.5 p.p, *p* < 0.05) than native graduates of equal social origin and performance level do. Moreover, the immigration-specific difference in educational choices significantly differs between social origin groups for both outcomes (each with *p* < 0.01). This supports our hypothesis about a larger immigration-specific difference in educational choices for immigrant graduates from low social origins than for graduates from high social origins (H2). Interestingly, we even observe less educational determination among immigrant graduates from high social origins compared to their native counterparts, which we did not expect. These results underscore the importance of investigating immigration-specific differences in educational choices for various social origin groups separately.

The interaction effect between social origin and immigrant status is accompanied by lower secondary effects of social origin within the group of graduates with immigrant backgrounds. Among native graduates, we observe strong secondary effects of social origin (for application and transition: 7 p.p.; Figures 3A,C). Among immigrant graduates, secondary effects of social origin are lower (for application and transition: 3 p.p.). Having moved the attention to the interaction between social origin and immigrant status, we can observe a further interesting result: Immigrant graduates from low social origins indeed apply more frequently to graduate programs and actually enroll slightly more often in the corresponding programs than native university graduates of the same social origin do. However, the application and transition behavior of these immigrants is, on average, not more ambitious than that of native graduates from high social origins (Figures 3A,C). This result is important for the question frequently discussed in scientific research regarding whether immigrants are potentially too ambitious (e.g., Tjaden and Hunkler, 2017). According to our results, it is both groups—immigrant graduates from low social origins and native graduates from high social origins—that are potentially overambitious or not.

Moving from the dichotomous differentiation between graduates with and without immigrant background to variations based on country of origin (Figures 3B,D), the picture is quite mixed. For some groups, immigration-specific differences in educational choices are remarkably large. For example, the difference reaches up to 6.4 percentage points for graduates with a background from Turkey from low social origins on submitting an application. Looking at variations depending on social origin, some groups of immigrant graduates descriptively show the expected pattern of a large immigration-specific difference on educational choices among graduates from low social origins and a comparatively small immigration-specific difference among graduates from high social origins. These groups are graduates of Turkish and Polish origin and those with a background from the residual country category. However, the differences based on social origin are not statistically significant for these country-specific immigrant groups, partly due to small sample sizes. Only for graduates from the former Soviet Union, the difference between social origin groups is indeed significant (for application: *p* < 0.01; for transition: *p* < 0.01). However, graduates from this group from high social origins unexpectedly make less ambitious educational choices than native graduates. The results for graduates from other labor market recruiting countries are even more unexpected: descriptively, these graduates are more ambitious than their native counterparts are when they are from high social origins, while they are less ambitious when they are from low social origins.

This mixed picture is reflected by the fact that the secondary effects of social origin differ remarkably between immigrant groups defined by country of origin (Figures 3A,C). Most groups of immigrants show comparable or slightly lower secondary effects of social origin than native graduates do (Turkey, Poland, other countries). However, for the group from the former Soviet Union, the secondary effect of social origin completely diminishes. For the group from other labor market recruiting countries, the secondary effect is strongly increased.

Regarding lower probabilities of transforming applications into actual transitions, we did not explicitly test for differences between application and transition for each social group, since the sample sizes are partly insufficient. However, smaller immigration-specific differences in the actual transition than for the application are descriptively visible for every social group (Figures 3B,D). This suggests that all immigrant groups have greater difficulties in realizing their plans than natives.

In summary, the results support our expectation of an interaction between immigrant status and social origin in predicting educational choices (H2). While immigrant graduates from low social origins prove to make choices that are more ambitious than their native counterparts do, this is not true for immigrants from high social origins. Looking at country-specific variations, the predicted pattern of an interaction between social origin and immigrant status (H2) holds for persons with a background from Turkey, Poland, and other countries, even though it does not reach statistical significance. For university graduates with a background from the former Soviet Union and from former labor market recruiting countries, the results are unexpected, with one group even showing reversed results.

## 5. Summary and discussion

In our study, we investigated whether the pattern of stronger educational determination among immigrants holds true after obtaining the first university degree. Against the background of research on earlier educational stages, we examined whether immigrants make more ambitious educational choices also at the transition to graduate programs compared to natives, net of social origin and academic performance.

Our results show that some immigrant groups have a stronger determination for graduate studies than their native counterparts. However, we also observe strong variations in the immigration-specific difference. Under certain conditions, the difference is quite large. It reaches up to 6.4 p.p. (for the application behavior of graduates with a Turkish background from low social origins) and thereby approximates the secondary effect of social origin (7 p.p.). Under other conditions, we find no immigration-specific difference, and in some cases, we even observe natives to make more ambitious choices than immigrants. We uncovered three sources of variation, thereby contributing more broadly to research on immigration-specific disparities in education.

First, we see strong variations based on *social origin*. As we have expected (H2), immigrants from low social origins are more ambitious than native graduates from equal social origins, whereas the immigration-specific difference in choices among graduates from high social origins is considerably smaller. This interaction between social origin and immigrant status is in line with previous studies on early and middle educational stages (Dollmann, 2017; Sudheimer and Buchholz, 2021).

As these results provide insights on educational pathways within higher education, they may stimulate theory development. Regarding the choices of immigrants, researchers have not yet specified how strong their desire for upward mobility is. More specifically, we are not aware of elaborated ideas on what distance from parents in terms of socioeconomic positioning immigrants strive for. Our results suggest that immigrants seek *status maximization* – a theoretical idea that we understand as a specification of the motivation for status gain. Individuals from low social origins would most likely experience upward mobility with a bachelor’s degree. However, most immigrant graduates from low social origins still strive for further education after having graduated from bachelor’s programs, which suggests that they might strive for status maximization against the background of already accomplished educational successes. This theoretical perspective would underscore the significance of the question of how far-reaching the stronger determination of immigrants from low social origins is, i.e., whether it persists in extremely high educational stages and professional careers even if the individuals are from low social origins.

Furthermore, our results on the interaction between social origin and immigrant status provide two interesting side results. Regarding the strength of the educational determination, we do not observe immigrant graduates from low social origins to make more ambitious educational choices than natives from high social origins, net of academic performance. Therefore, it might be sensible to adjust the perception of immigrant individuals being potentially overambitious (e.g., Tjaden and Hunkler, 2017) if the observed pattern also holds for other educational stages and contexts. Our results suggest that a research perspective that renders the strong educational determination of immigrants as being potentially problematic should at least be expanded to include the educational choices of natives from high social origins. This change in perspective might also stimulate research on educational choices of native students from low social origins, which could also benefit from a comparison with immigrant students from low social origins when it comes to explaining or addressing their motives (*cf.* Pietrzyk et al., 2023). Furthermore, we replicated previous findings of stronger secondary effects of social origin for natives than for immigrants (e.g., Relikowski et al., 2010).

Second, we have uncovered the *country of origin* as being significant for variations of the immigration-specific difference in educational choices. Against the background of former research, we have expected that persons with a background from Turkey would be the most ambitious of all immigrant groups (H3). However, this is not the case. Persons with a background from Poland and from countries of the residual category are as interested in further education as persons with a background from Turkey, especially when they are from low social origins. In contrast, graduates with a background from the former Soviet appear to be less determined to continue their education than native persons, especially those from high social origins.

The pattern of country-specific variations underscores the importance of further research on its theoretical explanations, which we could not carry out. However, given our findings, we strongly suggest testing explanations based on socioeconomic conditions, circumstances of immigration and experiences within the host country before moving to cultural explanations. The finding that graduates with a Polish background, who did not receive much attention in previous research due to insufficient data, have a similar level of determination as graduates with a background from Turkey (at least when they are from low social origins) suggests that explanations touching on cultural factors might frequently not hold true.

Third, we have expected that the investigation of educational choices would lead to different results depending on whether we examined the *application* to graduate programs or the *actual transitions* (H4). We see our expectations to be accurate since we constantly observe higher immigration-specific differences for applications than for actual enrollment. This observation highlights the importance of considering that transitioning to graduate studies is influenced not only by the desire for further education but also by external selection. Particularly in education systems that are less choice-driven and more performance-based than it is the case in Germany, the gap between applications and actual transitions could be even more pronounced. For example, graduate enrollment rates are considerably lower in the UK or the US (see Tienda and Zhao, 2017; Wakeling and Laurison, 2017). By indirectly framing disparities in education as a series of distinct stages, our approach enabled a description of the challenges that specific groups of graduates may encounter when translating their ambitions into actual transitions.

Building upon the gap between applications and transitions, we showed that immigrants have fewer chances of transforming their applications into actual transitions than natives. Immigrants’ lower performance levels (compared to natives from equal social origins) were associated with these lower chances. However, further factors that we could not consider may be influential (*cf.* Scheller et al., 2013; Alesi and Neumeyer, 2017, 85f). First, stronger difficulties in accessing information among immigrant students, based on their, on average, lower social integration within universities, might also be responsible for lower admission rates. Second, some graduates might not have enrolled in programs to which they have been admitted due to various reasons like financial constraints. Further research should delve deeper into these potential factors playing a role in the fewer chances of transforming aspirations into enrollment of immigrant graduates.

Finally, our study suffers from several limitations. First, we only observed applications and transitions within a time frame of 1.5 years after obtaining a bachelor’s degree. At first glance, this period seems sufficient, as delayed transitions are rare (Briedis et al., 2016, 8f). However, this could change in the long term if more graduates upgrade their first degree after a few years of work experience, as intended in the framework of the Bologna reform, and if upgrading is more tightly integrated into professional career paths. Second, we assumed that the interaction between social origin and immigrant background is caused by differential motives for status maintenance and status gain. Our results are consistent with what the theory of different motives predicts. However, they do not allow us to conclude whether the immigration-specific difference in educational choices is indeed due to immigrants’ striving for status advancement or whether, alternatively, it results from other factors, such as immigrants’ aspiration for relative status maintenance (Ichou, 2014; Feliciano and Lanuza, 2017; Engzell, 2019; Tong and Harris, 2021). Third, we focused on parental educational attainment when capturing social origin instead of applying a broader concept of social origin, which explicitly considers parents’ labor market positions. This might be sensible within Germany, where parental educational attainment strongly influences educational pathways. In other contexts, where, for example, high tuition fees are charged and, therefore, parents’ wealth is more important (e.g., the UK or the US), such an operationalization might be insufficient.

However, even against the backdrop of these limitations, we can say that also within higher education some groups of immigrant graduates strive more determinately for further education than native graduates of equal social origin and performance level do. We hope that future research on immigration-specific disparities in education will expand upon our observations by delving further into whether immigrants truly seek to maximize their social status in light of their existing achievements, by moving to immigrant groups that did not receive much research attention thus far, such as persons with a Polish background, and by elaborating more deeply which steps students need to take before actual enrollment and how chances for taking these steps differ between different social groups. Further, it remains the subject of upcoming research whether and under what conditions immigrants’ bold educational choices translate into completion of master’s programs and high labor market positions.

## Data availability statement

The data analyzed in this study is subject to the following licenses/restrictions: To get access to the KOAB data, researchers can contact the International Centre for Higher Education Research (INCHER) in Kassel, Germany. Requests to access these datasets should be directed to INCHER-Kassel: https://www.uni-kassel.de/forschung/en/incher/kontakt.

## Ethics statement

Ethical approval was not required for the studies involving humans because it was not legally required for this study. The studies were conducted in accordance with the local legislation and institutional requirements. The participants provided their written informed consent to participate in this study.

## Author contributions

All authors listed have made a substantial, direct, and intellectual contribution to the work and approved it for publication.

## Footnotes

^1^The focus on parents’ educational attainment instead of applying a broader concept of social origin that explicitly considers the labor market position of the parents might, however, *underestimate* the high educational ambitions of immigrant students, if the formal certificates of immigrant students’ parents have not been fully recognized in the host country’s labor market.

^2^In Germany, master’s programs were introduced during the Bologna reform beginning at the turn of the current century with master’s certificates largely corresponding to the former long-cycle degrees (Diploma and Magister). Therefore, the scenario description of parents holding master’s degrees for the German case could be rephrased into parents holding Diploma or Magister degrees.

^3^The project was carried out jointly by the International Centre for Higher Education Research (INCHER) at the University of Kassel and higher education institutions in German-speaking countries for research and quality assurance purposes. Information on the research design and the questionnaire are available in Heidemann and Janson (2009) and Pietrzyk and Graser (2017).

^4^We assess the time period of 1.5 years as being sensible for investigating our research questions since it measures the direct application for and transition to master’s programs, respectively. Therefore, the captured educational aspirations can be assumed to be not much affected by experiences in the labor market. Changes in aspirations driven by labor market experiences of university graduates would be an interesting research question in its own right. However, when longer periods are observed, the transition rate increases only slightly from 67% after 1.5 years to 72% after 6.5 years (Briedis et al., 2016, 8f).

^5^We excluded older graduates to homogenize the sample. When university graduates reach a certain age, educational aspirations might be additionally driven by the consideration of whether further investments in education are sensible given an expected short time remaining in the labor market. Therefore, graduates might assume that continuing education might play a smaller role in status acquisition. Details on the data selection process are reported in Supplementary Table S1.

^6^Detailed information on the technical implementation of the imputation is provided in Supplementary Table S2.

^7^Supplementary Tables S3–S5 provide details on instruments and operationalization steps.

^8^We are not able to distinguish between the first and the second generation for all respondents included into our analyses. However, for the graduating class of 2014 a differentiation is available. Within this class, 39.3% of all immigrant graduates are first-generation immigrants (i.e., they are born abroad) and 60.7% are second generation immigrants (i.e., at least one parent is born abroad, while the respondent is born in Germany). First-generation immigrants are most prevalent among graduates with an immigrant background from the former Soviet Union (85.1%). For all other immigrant groups differentiated by country of origin, the share of first-generation immigrants varies between 15.7 and 29.0%.

^9^Before the Bologna Reform, the German higher education system was characterized by a sharp distinction between universities of applied sciences (West Germany) and engineering schools (East Germany) on the one hand and traditional universities on the other hand. Studies at traditional universities typically had a longer standard period of study. Furthermore, bachelor’s degrees fall into the short-cycle category. However, due to the late introduction of bachelor’s programs in Germany, parents of the respondents did not obtain bachelor’s degrees when they got their education in Germany. For graduates whose parents attended university abroad and, therefore, potentially graduated from a bachelor’s program, the KOAB data is not perfectly suited since it does not cover the category “bachelor’s degree” for parent’s education explicitly. Therefore, some respondents, presumably those with an immigrant background, might have incorrectly classified their parents as holding a long-cycle university degree when their parents had in fact obtained a bachelor’s degree, i.e., a short-cycle degree. We assess this noise as being neglectable, since such a misclassification presumably leads to an *underestimation* of immigration-specific differences in educational choices due to the potentially falsely assigned higher levels of social origin among immigrant graduates.

^10^The parents of the respondents typically obtained Diploma or Magister degrees at traditional universities when following into this category, again, due to the late introduction of bachelor’s and master’s programs.

^11^See Supplementary Figures S1, S2.

^12^Distributions of variables by immigrant groups are reported in Supplementary Table S6.

^13^As additional controls, all models contain field of study, gender, and the year of graduation. Furthermore, we conducted the regressions with robust standard errors based on a cluster variable reflecting the institution at which the respondent obtained the bachelor’s degree and the year of graduation.
