# Supplementary material for: Done with a degree? Immigration-specific disparities among holders of bachelor’s degrees in the transition to graduate studies in Germany
Source: Front Sociol. 2023 Oct 12;8:1204164. doi: 10.3389/fsoc.2023.1204164 (PMC10602756; doi:10.3389/fsoc.2023.1204164)
Supplement: Supplementary file 1 [file Data_Sheet_1.pdf]

frontiers

Supplementary Material

Supplementary Table S1. Data selection steps.

Subsamples and excluded graduates	*N*	*U*	*UaS*
Holders of bachelor's degrees from German higher education institutions, graduation classes 2011-2014	90,556	49	32
Excluded: at least one relevant question is not asked in the questionnaire of a specific institution or to a specific subgroup	8,680	5	1
Remaining sample:	81,876	44	31
Excluded: higher education entry qualification obtained abroad (or missing information)	1,955		
Remaining sample:	79,921	44	31
Excluded: persons older than 45 years	411		
Remaining sample:	79,510	44	31
Excluded: cases with missing values on outcome variables	8,766		
Analysis sample: holders of bachelor's degrees from German higher education institutions, graduation classes 2011-2014; all relevant questions asked; higher education entry qualification obtained in Germany; maximum age at graduation 45 years; valid answers on outcome variables.	70,744	44	31

Notes: N number of graduates; U number of traditional universities; UaS number of universities of applied sciences (*Fachhochschulen*).

Supplementary Table S2. Imputation model specifications.

Included variables	Imputation model method	Further specifications
Outcome variables		
Application	Logistic	Conditional on: transition to further program (no transition)
Transition	Logistic	
Independent variables		
Immigrant background	Multinomial	
Social origin	PMM	Conditional on: immigrant background group (for each group)
Control variables		
School GPA (standardized)	PMM	
GPA in bachelor's program (standardized)	PMM	
Field of study	-	
Gender	Logistic	
Year of graduation	-	
Auxiliary variables		
Age at graduation from bachelor's program (20-45)	PMM	
Vocational education before bachelor's program (0/1)	Logistic	
Alternative indicator of enrollment (immediately after graduation from bachelor's program or 1.5 years after graduation) (0/1)	Logistic	
Indicator of labor market entry or job search immediately after graduation from bachelor's program (0/1)	Logistic	
Study satisfaction with bachelor's program (1-5)	PMM	
Evaluation of contacts to fellow students in bachelor's program (1-5)	PMM	
Reason for no transition: "Job offer" or "wish to gain work experience" (0/1)	Logistic	Conditional on: transition (no transition)
Reason for no transition: "No admission to desired program" (0/1)	Logistic	Conditional on: transition (no transition)

Notes: PMM = predictive mean matching; multinomial = multinomial logistic regression; - = no missing values. More information about the imputation is provided on the next page.

General approach:

- Iterated chained equations
- All model variables, including interaction between immigrant background and social origin
- Auxiliary variables
- Imputation of outcomes, then deletion of cases with imputed outcomes
- Number of imputed data sets M = 30

Handling of interaction:

- Conditional imputation of social origin, separate for each immigrant group
- Conditional social origin variables are also used to impute other variables (outcome variables, achievement etc.)

Overlapping outcomes and conditional imputations:

- Applications and transitions overlap partially and can be differentiated into three groups (no application, application without transition, application with transition).
- The most natural way to impute data would be to first impute application and to subsequently impute transition for the subgroup with applications.
- However, there are more missing values on the application variable than on the transition variable (as the questions on applications are filtered based on the same questions that were used to construct the transition variable).
- Therefore, we first imputed the transition variable and then imputed the application variable for the subgroup without transition.
- With this procedure, a few cases with valid information on the application variable and missing information on transitions (this may be caused by going back and forth in the online questionnaire; n = 105/79510 or 0.1%) are practically set to missing to facilitate the conditional imputation. After the imputation of missing variables, imputed values of applications are corrected to their valid values.
- Similarly, some graduates had missing values with regards to immigrant background, but valid information on social origin (n = 102/79510 or 0.1%). These values are set to missing to facilitate conditional imputation. After imputation, some values are corrected back to their valid values.
- Further checks with reduced numbers of imputed datasets showed that neither the direction of conditional imputation nor the correction of unnecessary imputed values leads to altered results.

Supplementary Table S3. Operationalizations of outcomes.

Variable	Questions	Operationalization
Transition	[2011–2013]<br>Have you pursued further studies after your bachelor's degree?<br><br><br><br><br>[2014]<br>Did you pursue a master's program after your bachelor's degree?<br><br>Did you take up another course of study after your bachelor's degree, but it is not a master's program?	[2011–2013]<br>1=yes,<br>if enrollment in further studies<br><br>0=no,<br>if not<br><br>[2014]<br>1=yes,<br>if enrollment in master’s program or other program.<br><br>0=no,<br>if both no
Application	How many times did you apply for further study and how many acceptances did you receive?<br><br>(5 Answer fields:<br>Number of applications at BA granting institution;<br>Number of applications at other institution;<br>Number of institutions to which one has applied;<br>Number of admissions at BA granting institution;<br>Number of admissions at other institution;<br><br>The questions were filtered, if a graduate never had the intention to enroll in a further program)	1=yes,<br>if at least one application at the BA institution or another institution.<br><br>or<br>if a transition was made without reported applications<br><br>or<br>if an admission was received without reported applications<br><br>0=no<br>if no application was reported<br><br>or<br>if the question was filtered (graduate never had the intention)

Notes: If question wording and operationalization varies between years, corresponding years are shown in square brackets. Regarding the transition in a further program, for 67.8% of those graduates who transitioned in a further program, additional details on the type of program are available. Among these graduates, 93.7% have enrolled in a master’s program.

Application variable: Data plausibilization steps

To create a dummy indicator for the application to further studies, three items on the number of applications were utilized to compute an unplausibilized variable (step 1) and these values were plausibilized with the number of admissions to further programs and with the transition variable itself (step 2). Lastly, filtering of the questions on applications is taken into account (step 3).

1) The unplausibilized application variable

- is based on
    - the number of applications at the BA granting institutions,
    - the number of applications at other institutions,
    - the number of different institutions to which a graduate has applied.
- takes the value
    - 0 (no application),
        - if all values are 0
        - or if at least one value is 0 and all other values are missing (item non-response)
    - 1 (application)
        - if at least one valid non-zero value of applications is available

2) The unplausibilized application variable is plausibilized as follows:

- missing values are set to 1 and zero-values are corrected to 1
    - if at least one valid non-zero value of the number of admissions (to either programs at the BA granting institutions or other institutions) is available
    - or if the graduate made a transition to a further program
- This step is done since graduates who have transitioned to a further program or were admitted to a further program must have applied to a further program.

3) Finally, missing values of the plausibilized application variable are set to 0

- if a graduate never had the intention to enroll in a further program and thus the questions about applications and admissions were filtered.

Supplementary Table S4. Operationalizations of independent variables.

Variable	Questions	Operationalization
Immigrant background	In which country were your parents born?<br><br>(separate answers for mother and father)	Answers were recoded into country categories.<br><br>0=both parents were born in Germany;<br>1=at least one parent was born in Turkey;<br>2=at least one parent was born in another labor market recruiting country (Italy, Greece, Portugal, Spain, countries of the former Yugoslavia);<br>3=at least one parent was born in a country of the former Soviet Union;<br>4=at least one parent was born in Poland;<br>5=at least one parent was born in another country;<br><br>Values are set to missing, if both parents are from two different categories 1−5
Social origin	What is your parents' highest professional degree?<br><br>(separate answer categories for mother and father)	0=none (no degree; unknown; item nonresponse, if only for one parent)<br><br>1=vocational education and training/VET (Apprenticeship; Vocational or commercial school; Master craftsman's or technician's)<br><br>2=HE short-cycle (technical college (GDR); university of applied sciences, engineering school, commercial academy)<br><br>3=HE long-cycle / master’s degree level (college of art or music; university)<br><br>4=doctorate (doctorate; habilitation)<br><br>highest degree level of mother and father

Supplementary Table S5. Operationalizations of control variables.

Variable	Questions	Operationalization
Achievement: School GPA	What was your grade point average on your higher education entrance qualification?<br><br>(range from 1.0=very good to 4.0=sufficient)	answers are<br>a) z-standardized<br><br>b) reversed (so that higher values indicate better grades)<br><br>One unit of the z-standardized scale corresponds to 0.62 units of the raw variable with values from 1.0 (very good) to 4.0 (sufficient).
Achievement: Study GPA	What was your final or average grade in this program?<br><br>(range from 1.0=very good to 4.0=sufficient)	answers are<br>a) z-standardized within combinations of graduation classes and fields of study (see below)<br><br>b) reversed (so that higher values indicate better grades)<br><br>Field-specific means range between 1.75 and 2.33 on the raw scale from 1.0 (very good) to 4.0 (sufficient).<br>One unit of the z-standardized scale corresponds to values ranging from 0.34 to 0.54 units of the raw variable, depending on the field of study and the year.
Field of study	In which field of study/ degree program did you study?<br><br>(answers are available in the field of study classification of the Federal Statistics Office: 'Studienbereichsgliederung', Version from 2011)<br><br>What degree have you obtained? (only to differentiate bachelor's degree in teaching from other Bachelor's degrees)<br><br>Additionally, information on the type of higher education institution is included (U=university, UaS=university of applied sciences)	1=U Math/Sciences (436,437,439−444)<br>2=U Engineering (757−760,861−870)<br>3=U Computer Science (438)<br>4=U Economics (incl. Econ. Engineering; 330,331)<br>5=U Humanities/Arts (101−114,974−978)<br>6=U Social Sciences (115,324−326)<br>7=U Educ. Sciences (116,117)<br>8=U Social Work (327)<br>9=U Teaching (any field with BA degree in teaching)<br>10=U Other (all other fields from universities)<br><br>11=UaS Engineering (757−760,861−870)<br>12=UaS Computer Science (438)<br>13=UaS Economics (incl. Econ. Engineering; 330,331)<br>14=UaS Social Work (327)<br>15=UaS Other (all other fields from universities of applied sciences)
Gender	What is your gender?	0=male<br>1=female

Supplementary Table S6. Descriptive statistics by immigrant groups.

	Natives	Turkey	oLMC	fSU	Poland	Other
Application						
no	26.9 %	33.5 %	30.7 %	33.2 %	26.7 %	24.0 %
yes	73.1 %	66.5 %	69.3 %	66.8 %	73.3 %	76.0 %
Transition						
no	29.9 %	39.4 %	37.0 %	38.3 %	30.6 %	28.5 %
yes	70.1 %	60.6 %	63.0 %	61.7 %	69.4 %	71.5 %
Transition, if application = yes:						
no	4.0 %	9.0 %	9.1 %	7.8 %	5.3 %	5.9 %
yes	96.0 %	91.0 %	90.9 %	92.2 %	94.7 %	94.1 %
Social origin						
No degree	0.4 %	46.9 %	20.7 %	3.6 %	1.4 %	8.9 %
VET	48.7 %	39.4 %	53.8 %	43.3 %	63.7 %	36.0 %
Short-cycle HE	17.4 %	3.8 %	6.0 %	15.2 %	10.2 %	9.1 %
Long-cycle HE	26.8 %	8.5 %	15.5 %	35.1 %	22.3 %	36.4 %
Doctorate	6.7 %	1.3 %	3.9 %	2.9 %	2.3 %	9.6 %
School GPA	0.04	−0.64	−0.19	−0.11	−0.22	−0.04
	(1.00)	(0.89)	(1.01)	(0.96)	(1.00)	(1.03)
GPA in bachelor's program	0.05	−0.54	−0.22	−0.20	−0.17	−0.15
	(0.99)	(1.02)	(1.02)	(0.98)	(1.00)	(1.04)
Field of study						
U Math/Sciences	11.1 %	5.9 %	8.9 %	8.8 %	10.1 %	11.3 %
U Engineering	8.9 %	7.2 %	7.5 %	7.0 %	7.9 %	9.5 %
U Computer Science	2.2 %	2.9 %	2.1 %	2.3 %	2.2 %	2.9 %
U Economics	6.8 %	8.6 %	7.1 %	8.0 %	7.0 %	8.1 %
U Humanities/Arts	8.6 %	7.4 %	14.0 %	6.6 %	9.9 %	11.2 %
U Social Sciences	4.4 %	3.6 %	5.5 %	3.6 %	4.3 %	5.4 %
U Educ. Sciences	2.1 %	3.1 %	1.3 %	1.5 %	2.2 %	2.1 %
U Social Work	1.0 %	1.3 %	1.0 %	0.9 %	1.2 %	0.6 %
U Teaching	3.0 %	2.9 %	1.8 %	1.5 %	2.6 %	2.0 %
U Other	2.5 %	1.2 %	1.8 %	1.5 %	1.2 %	2.2 %
UaS Engineering	21.0 %	20.2 %	15.7 %	26.7 %	19.8 %	15.1 %
UaS Computer Science	2.9 %	5.2 %	2.8 %	4.3 %	4.7 %	3.1 %
UaS Economics	11.7 %	15.4 %	19.5 %	14.0 %	11.9 %	11.9 %
UaS Social Work	5.3 %	6.8 %	4.1 %	5.2 %	5.4 %	5.7 %
UaS Other	8.6 %	8.2 %	6.9 %	7.9 %	9.7 %	9.0 %
Gender						
Male	50.3 %	52.8 %	50.8 %	50.4 %	50.6 %	49.7 %
Female	49.7 %	47.2 %	49.2 %	49.6 %	49.4 %	50.3 %
Graduation class						
2011	24.5 %	21.7 %	24.9 %	22.3 %	21.9 %	23.0 %
2012	23.9 %	22.9 %	22.9 %	22.6 %	23.0 %	22.4 %
2013	26.2 %	28.7 %	26.9 %	27.9 %	27.4 %	26.0 %
2014	25.3 %	26.6 %	25.3 %	27.2 %	27.7 %	28.6 %

Notes: Shares (of categories) and means and standard deviations (of metric variables) are reported. Weighted and imputed data. (N=70,744).

Supplementary Table S7. Results of logistic regressions (immigration-specific differences across different levels of social origin).

**Outcome variable**	**Transition**		**Application**		**Transition**		**Application**	
	logit	p	logit	p	logit	p	logit	p
Immigrant background (pooled, Ref.: Natives)	−0.003		0.101	**				
Country-specific immigrant group (Ref.: Natives)								
Turkey					0.100		0.235	*
Other labor market recruiting countries (OLMC)					−0.166	+	0.015	
Former Soviet Union (FSU)					−0.191	**	−0.091	
Poland					0.195	**	0.246	**
Other					0.101	+	0.209	**
Social origin (Ref.: no degree)								
VET	−0.103		−0.123		−0.087		−0.080	
short-cycle HE degree	0.085		0.087		0.105		0.135	
long-cycle HE degree	0.285	**	0.278	*	0.304	**	0.325	**
doctorate	0.527	**	0.571	**	0.540	**	0.610	**
Achievement:								
School GPA	0.117	**	0.123	**	0.119	**	0.125	**
GPA in bachelor’s program	0.461	**	0.420	**	0.460	**	0.420	**
GPA in bachelor’s program (squared)	−0.076	**	−0.069	**	−0.076	**	−0.070	**
Field of study (Ref.: U Math/Sciences)								
U Engineering	−0.627	**	−0.610	**	−0.629	**	−0.611	**
U Computer Science	−1.483	**	−1.523	**	−1.483	**	−1.524	**
U Economics	−1.882	**	−1.849	**	−1.881	**	−1.848	**
U Humanities/Arts	−1.184	**	−1.166	**	−1.185	**	−1.168	**
U Social Sciences	−0.972	**	−0.880	**	−0.973	**	−0.881	**
U Educ. Sciences	−1.981	**	−2.001	**	−1.986	**	−2.006	**
U Social Work	−3.644	**	−3.392	**	−3.645	**	−3.392	**
U Teaching	0.077		0.065		0.076		0.064	
U Other	−1.768	**	−1.684	**	−1.767	**	−1.684	**
UaS Engineering	−2.851	**	−2.796	**	−2.847	**	−2.790	**
UaS Computer Science	−3.053	**	−2.978	**	−3.054	**	−2.977	**
UaS Economics	−3.391	**	−3.283	**	−3.388	**	−3.281	**
UaS Social Work	−4.233	**	−4.008	**	−4.236	**	−4.010	**
UaS Other	−2.932	**	−2.838	**	−2.934	**	−2.839	**
Gender (Ref.: male)								
female	−0.178	**	−0.179	**	−0.177	**	−0.178	**
Graduation class (Ref.: 2011)								
2012	−0.027		−0.013		−0.027		−0.013	
2013	−0.066		−0.056		−0.065		−0.055	
2014	−0.018		−0.012		−0.018		−0.012	
Constant	3.357	**	3.464	**	3.338	**	3.419	**

Notes: + $p < 0.10$, * $p < 0.05$, ** $p < 0.01$; U = University, UaS = University of Applied Sciences.

Supplementary Table S8. Results of logistic regressions (immigration-specific differences at different levels of social origin).

**Outcome variable**	**Transition**		**Application**		**Transition**		**Application**	
	logit	p	logit	p	logit	p	logit	p
Immigrant background (pooled, Ref.: Natives)	−0.058		−0.029					
Country-specific immigrant group (Ref.: Natives)								
Turkey					−0.115		−0.096	
Other labor market recruiting countries (OLMC)					−0.230		−0.248	
Former Soviet Union (FSU)					−0.484		−0.341	
Poland					0.296		0.105	
Other					0.374		0.463	
Interactions with social origin:								
Immig. (pooled) # VET	0.156		0.222					
Immig. (pooled) # short-cycle HE	−0.035		0.075					
Immig. (pooled) # long-cycle HE	−0.101		−0.032					
Immig. (pooled) # doctorate	−0.001		0.156					
Turkey # VET					0.407		0.511	
Turkey # short-cycle HE degree					0.488		0.537	
Turkey # long-cycle HE degree					0.368		0.477	
Turkey # doctorate					−1.274	+	0.510	
OLMC # VET					0.127		0.313	
OLMC # short-cycle HE degree					−0.549		0.132	
OLMC # long-cycle HE degree					0.340		0.637	
OLMC # doctorate					−0.820		−0.800	
FSU # VET					0.449		0.400	
FSU # short-cycle HE degree					0.298		0.268	
FSU # long-cycle HE degree					0.063		−0.002	
FSU # doctorate					0.737		0.886	
Poland # VET					−0.040		0.171	
Poland # short-cycle HE degree					−0.086		0.152	
Poland # long-cycle HE degree					−0.274		0.059	
Poland # doctorate					−0.523		−0.067	
Other # VET					−0.241		−0.216	
Other # short-cycle HE degree					−0.395		−0.365	
Other # long-cycle HE degree					−0.414		−0.419	
Other # doctorate					−0.174		−0.208	
Educational origin (Ref.: no degree)								
VET	−0.167		−0.249		−0.168		−0.251	
short-cycle HE degree	0.047		−0.019		0.046		−0.020	
long-cycle HE degree	0.267		0.196		0.266		0.195	
doctorate	0.488	*	0.456	*	0.487	*	0.455	*
Achievement:								
School GPA	0.118	**	0.124	**	0.119	**	0.126	**
GPA in bachelor’s program	0.461	**	0.420	**	0.461	**	0.420	**
GPA in bachelor’s program (squared)	−0.075	**	−0.069	**	−0.077	**	−0.070	**

Outcome variable	Transition		Application		Transition		Application	
	logit	p	logit	p	logit	p	logit	p
Field of study (Ref.: U Math/Sciences)								
U Engineering	−0.628	**	−0.610	**	−0.630	**	−0.611	**
U Computer Science	−1.481	**	−1.522	**	−1.483	**	−1.524	**
U Economics	−1.882	**	−1.849	**	−1.883	**	−1.850	**
U Humanities/Arts	−1.184	**	−1.166	**	−1.185	**	−1.167	**
U Social Sciences	−0.972	**	−0.879	**	−0.973	**	−0.878	**
U Educ. Sciences	−1.981	**	−2.002	**	−1.988	**	−2.007	**
U Social Work	−3.643	**	−3.391	**	−3.644	**	−3.393	**
U Teaching	0.076		0.064		0.077		0.064	
U Other	−1.768	**	−1.685	**	−1.766	**	−1.683	**
UaS Engineering	−2.851	**	−2.795	**	−2.847	**	−2.791	**
UaS Computer Science	−3.053	**	−2.977	**	−3.053	**	−2.977	**
UaS Economics	−3.390	**	−3.282	**	−3.389	**	−3.280	**
UaS Social Work	−4.233	**	−4.008	**	−4.238	**	−4.013	**
UaS Other	−2.932	**	−2.838	**	−2.937	**	−2.841	**
Gender (Ref.: male)								
female	−0.178	**	−0.179	**	−0.177	**	−0.178	**
Graduation class (Ref.: 2011)								
2012	−0.026		−0.012		−0.025		−0.012	
2013	−0.064		−0.054		−0.064		−0.054	
2014	−0.017		−0.012		−0.018		−0.012	
Constant	3.403	**	3.575	**	3.405	**	3.577	**

Notes: + $p < 0.10$, * $p < 0.05$, ** $p < 0.01$; U = University, UaS = University of Applied Sciences.

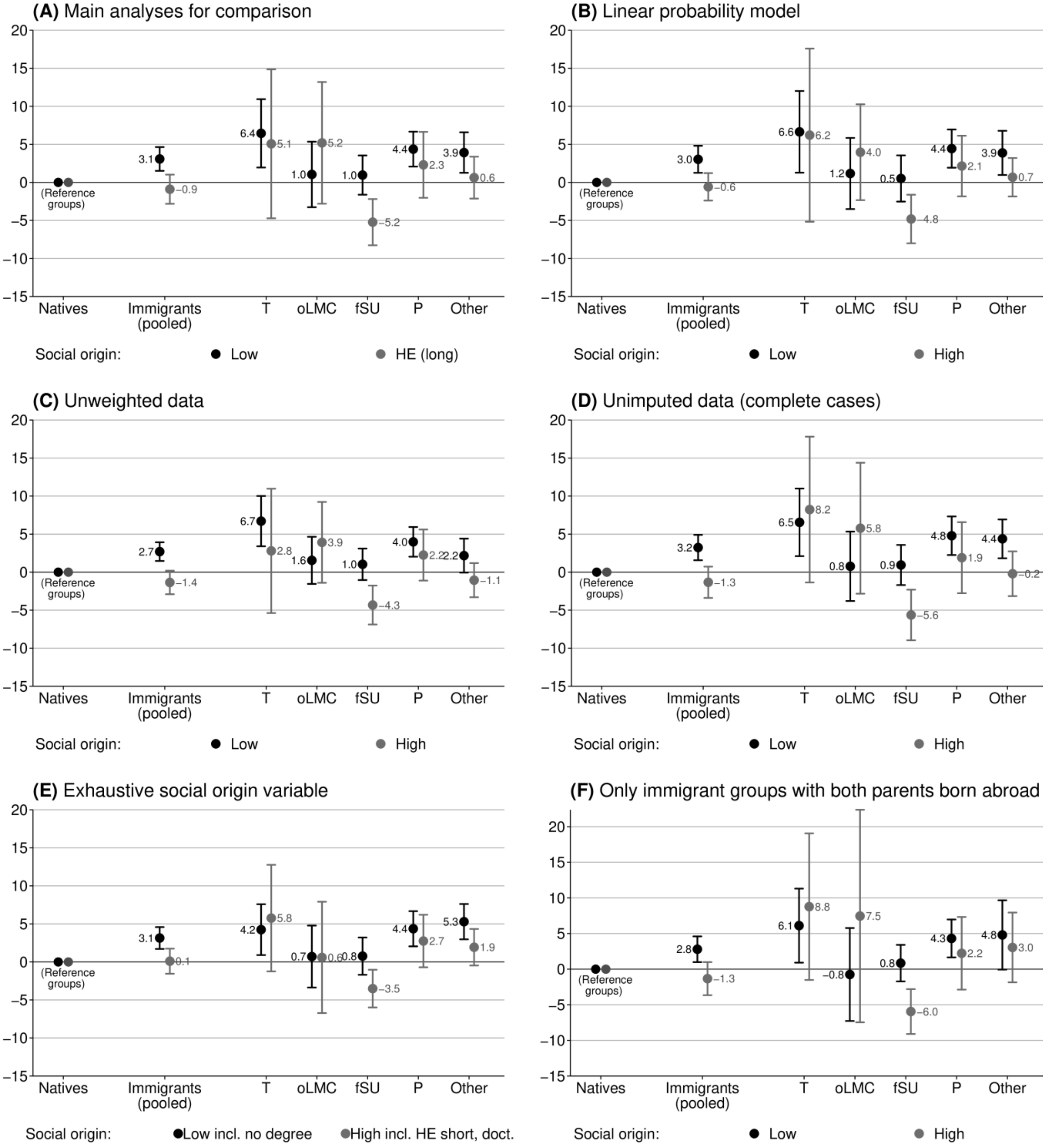

Supplementary Figure S1. Immigration-specific differences in application at different levels of social origin: alternative specifications.

Notes: Graph A illustrates the main results as reported in the main section in Figure 3B; graphs B to F show the results for alternative specifications. All graphs illustrate differences between immigrant and native bachelor's graduates of the same social origin in quantities of percentage point differences (AME × 100); 95% confidence intervals; controls: achievement, field of study, gender, and year of graduation. Immigrants (pooled): dichotomous operationalization of immigrant background regardless of country of origin; T: Turkey, oLMC: other labor market recruiting countries, fSU: former Soviet Union, P: Poland, Other: other countries.

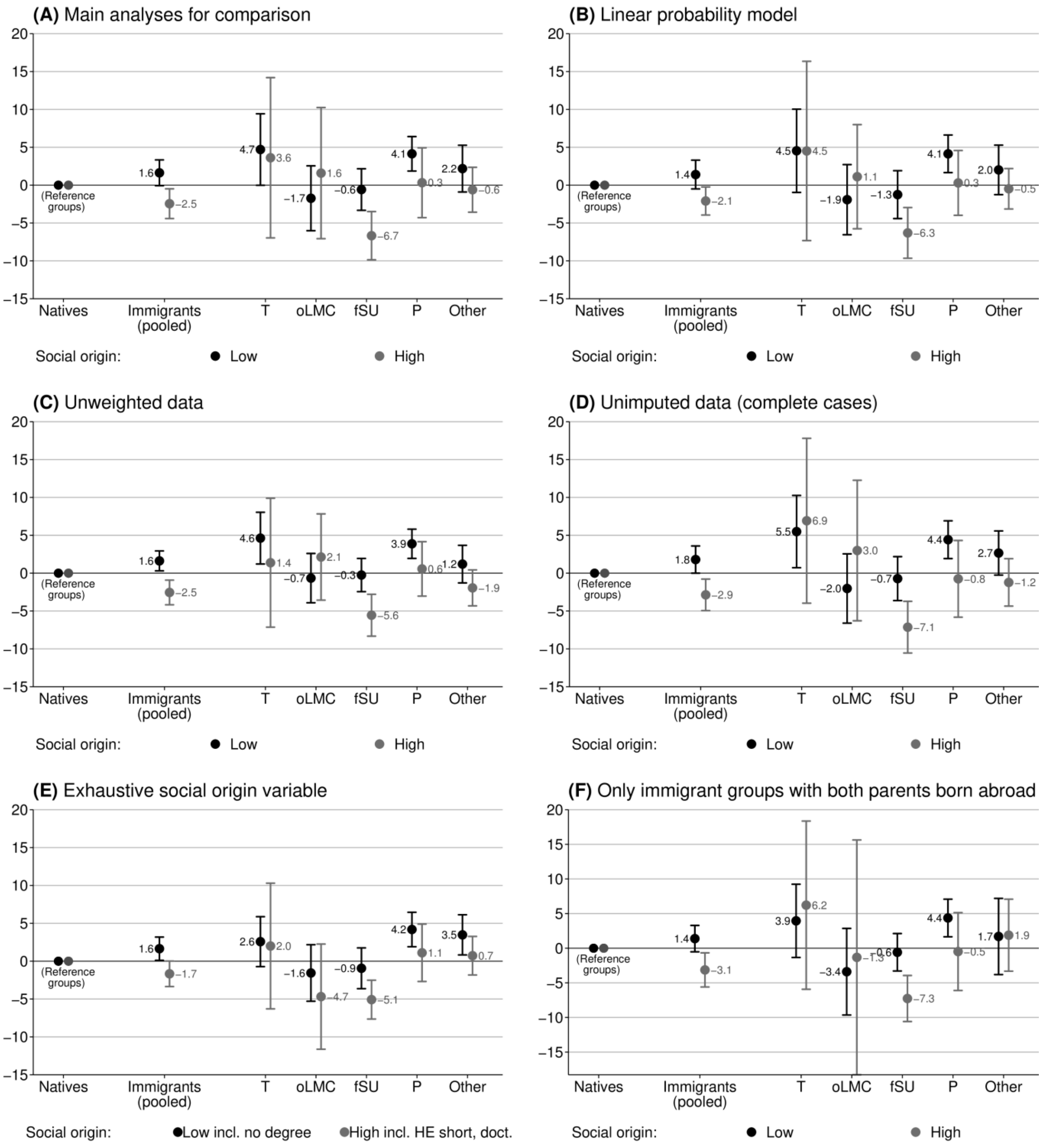

Supplementary Figure S2. Immigration-specific differences in transition at different levels of social origin: alternative specifications.

Notes: Graph A illustrates the main results as reported in the main section in Figure 3D; graphs B to F show the results for alternative specifications. All graphs illustrate differences between immigrant and native bachelor's graduates of the same social origin in quantities of percentage point differences (AME × 100); 95% confidence intervals; controls: achievement, field of study, gender, and year of graduation. Immigrants (pooled): dichotomous operationalization of immigrant background regardless of country of origin; T: Turkey, oLMC: other labor market recruiting countries, fSU: former Soviet Union, P: Poland, Other: other countries.

We examined the extent to which the main results are robust to alternative analytical procedures and alternative operationalizations of independent variables. More specifically, concerning *analytical procedures* we checked whether the results vary when we use linear probability models instead of logistic regressions, when we use no weights instead of weighting data and when we apply list-wise deletion instead of imputation. Additionally, *concerning the operationalization of independent variables* we checked whether the results differ when we apply a dichotomous and exhaustive operationalization of social origin based on whether at least one parent has graduated from university instead of focusing two levels of social origin (i.e., vocational training and long-cycle higher education) and when we exclude immigrant graduates who have only one parent that was born abroad instead of including these graduates into the category of immigrant graduates. Besides these outlined variations, we conducted the same analyses as described in the main section.[1]

The results of these robustness checks for the *application* are illustrated in Supplementary Figure 1 and for the *transition* in Supplementary Figure 2. For providing a convenient way of comparing the results from the main section with the robustness checks, we included the analysis from the main section into the illustration (Supplementary Figure 1A and Supplementary Figure 2A).

The illustrations neatly show that the results are largely robust to variations concerning the *analytical procedure*. More specifically, when applying a linear probability model (Supplementary Figure 1B and Supplementary Figure 2B) instead of logistic regression, the pattern of the immigration-specific differences does not change. However, some estimators differ slightly in terms of the effect sizes. The same is true for using unweighted data (Supplementary Figure 1C and Supplementary Figure 2C) instead of weighted data. The only exception is that the immigration-specific difference in application for persons with a background from other countries is not statistically significant anymore with unweighted data. Regarding the transition, the immigration-specific difference when a dichotomous measurement is applied reaches statistical significance with unweighted data. The results are also largely robust regardless of whether data is imputed or whether list-wise deletion is applied (Supplementary Figure 1D and Supplementary Figure 2D, respectively; $N = 58{,}135$). The direction and significance of the immigration-specific differences does not change for application, while a slight increase in the difference for graduates with a background from Turkey of low social origin leads to the difference in transition reaching statistical significance when using complete cases only.

Furthermore, the illustration reveals that the results are also largely robust to different *operationalizations* of independent variables. Concerning social origin, we used a measurement based on five levels and focused on two of these five categories when presenting the results (i.e., vocational training and long-cycle higher education). However, the results do not differ much when a dichotomous and exhaustive operationalization of social origin, which indicates whether at least one parent graduated from higher education, is applied (Supplementary Figure 1E and Supplementary Figure 2E). The direction and the significance of the immigration-specific difference remain largely stable under this specification. The only exceptions are that the differences in transition for immigrant graduates of

---

[1] That is, in estimating the immigration-specific differences in application and transition we calculated the difference between graduates with and without an immigrant background under consideration of the social origin and achievement level as well as of further background characteristics (field of study, gender and the year of graduation). Furthermore, we also applied cluster robust standard errors with a cluster variable reflecting the institution at which the bachelor's degree was attained and the year of graduation.

low social origin indicated by the dichotomous operationalization and for graduates with a background from the residual country category are slightly increased and reach statistical significance.

Concerning the operationalization of the immigrant background, we included graduates with one parent born abroad and the other parent born in Germany into the category of immigrant graduates. When this measurement is applied, around one third of the immigrant graduates have one parent that is born in Germany. The share is even higher within the group of graduates from high social origins (41.4% within the group whose parents graduated from long-cycle higher education vs. 31.8% for the group whose parents attended vocational training). These graduates with only one parent born outside Germany might exhibit relatively small immigration-specific differences, which in turn would lead to an attenuation of immigration-specific differences in our main analysis. Furthermore, the empirical association between social origin and the share of persons with only one parent born abroad might have driven the established interaction effects between social origin and immigrant status if immigration-specific differences were indeed smaller among graduates with only one parent born abroad.

To test whether graduates with both parents born abroad actually show larger immigration-specific differences than the results of our main analysis suggest, we excluded graduates with only one parent born outside Germany and rerun the analysis (Supplementary Figure 1F and Supplementary Figure 2F, respectively; N = 66,947 to 67,030, varying across imputations). Again, the direction and significance of the immigration-specific differences remain largely unchanged under this specification. However, for the application (Supplementary Figure 1F), the immigration-specific difference for graduates with a background from other countries from low social origins is not significant anymore even though the point estimator does not decrease. More importantly, some of the immigration-specific differences are descriptively larger when only graduates with both parents born abroad are included into the category of immigrant graduates. This is the case for graduates from high social origins with a background from Turkey and from other labor market recruiting countries. This leads to graduates with a Turkish background from low social origins descriptively no longer showing a larger immigration-specific difference than graduates with a Turkish background from high social origins. This finding supports the idea that the established interaction effect between social origin and immigrant status might be indeed driven by the association between social origin and the share of graduates with only one parent born abroad for this group of graduates with a Turkish background. Yet, the interaction effect between social origin and immigrant status descriptively still holds true for all other immigrant groups for which it was originally present. For the transition (Supplementary Figure 2F), with the alternative operationalization, we do not any longer observe larger immigration-specific differences for graduates from low social origins than among their peers from high social origins among graduates from Turkey and from other countries. Yet, the pattern of graduates from low social origins showing larger immigration-specific differences than graduates from high social origins descriptively still holds for the dichotomous operationalization and for graduates with a background from the former Soviet Union and from Poland.
